# Relationship between working hours and sleep quality with consideration to effect modification by work style: a community-based cross-sectional study

**DOI:** 10.1265/ehpm.23-00252

**Published:** 2024-03-20

**Authors:** Aya Yoshida, Keiko Asakura, Haruhiko Imamura, Sachie Mori, Minami Sugimoto, Takehiro Michikawa, Yuji Nishiwaki

**Affiliations:** 1 Department of Environmental and Occupational Health, Toho University Graduate School of Medicine, 5-21-16 Omori-Nishi, Ota-ku, Tokyo 143-8540, Japan; 2 Department of Preventive Medicine, School of Medicine, Toho University, 5-21-16 Omori-Nishi, Ota-ku, Tokyo 143-8540, Japan; 3 Department of Environmental and Occupational Health, School of Medicine, Toho University, 5-21-16 Omori-Nishi, Ota-ku, Tokyo 143-8540, Japan; 4 Graduate School of Health and Nutrition Sciences, The University of Nagano, 8-49-7 Miwa, Nagano City, Nagano 380-8525, Japan

**Keywords:** Sleep quality, Athens Insomnia Scale, Long working hours, Work style, Effect modification, Community-based study, Japan

## Abstract

**Background:**

Although longer working hours are associated with lower sleep quality, it is still necessary to work a certain number of hours to make a living. In this study, we investigated the relationship between working hours and sleep quality in a community setting. We then explored how to manage work style while maintaining the sleep quality of workers without markedly reducing working hours.

**Methods:**

4388 day-time workers in various occupations living in Ota ward in Tokyo were included in the analysis. The relationship between working hours and sleep quality measured by the Athens Insomnia Scale was examined by ANOVA and linear regression models. Effect modification by work style (work end time, shift in working start and end time, current work from home status, change in work place) on the relationship between working hours and sleep quality was investigated by multivariate linear regression models.

**Results:**

Longer working hours were significantly associated with lower sleep quality. The magnitude of the relationship between long working hours and low sleep quality was significantly larger when work end time was later (p for trend of interaction < 0.01) and when working start and end time were shifted later (vs no change, p for interaction = 0.03). The relationship was marginally greater when the proportion of work from home was increased (vs no change, p for interaction = 0.07).

**Conclusions:**

A relationship between longer working hours and lower sleep quality was observed among workers. Leaving work earlier or optimizing the work environment at home may diminish the adverse effect of long working hours on sleep quality.

**Supplementary information:**

The online version contains supplementary material available at https://doi.org/10.1265/ehpm.23-00252.

## Background

Sleep is a fundamental determinant of health, and sleep quality impacts people’s health and life. In a review, Ohayon [1] stated that nearly one-third of the general population experienced symptoms of insomnia and that this constituted a major public health problem. Poor sleep in both quality and quantity is known to cause obesity [2], hypertension [3], stroke and coronary disease [4], diabetes [5], and metabolic syndrome [6]. Sleep quality is also known to substantially impact mental health [7].

Long working hours are known to have adverse effects on people’s health, including sleep problems [8–11]. Bannai A et al. concluded that long working hours were associated with poor sleep condition regardless of variations in the measurement of sleep quality and the definition of long working hours across studies in their review paper [8]. A meta-analysis by Litwiller [12] indicated that poor sleep, in both quality and quantity, related to poorer health outcomes in workers, and led to poorer work performance, higher risk of work-related accidents, and higher tendency to resign. Similar studies have been primarily conducted in specific occupational groups, such as teachers [13, 14]; workers in a single organization, such as civil servants in a city [10]; male workers [15]; and workers in public safety sector [16]. In contrast, community-based studies which involve a large range of occupations are limited [17].

Sleep quality is associated not only with working hours, but also commuting time [14, 18, 19], shift work [20–22], and work time control [23]. Further, with the increasing number of employees who work from home due to the COVID-19 pandemic, a growing number of studies have shown an association between ‘work from home (WFH)’ and work productivity, work engagement, as well as worker’s mental state. However, studies evaluating the direct effect of WFH on sleep quality are limited and the results are inconsistent [24, 25].

According to OECD data [26], total working hours per year per worker (hr/yr/w) in Japan have decreased constantly since the 1980s, reaching 1607 hr/yr/w in 2021. This was shorter than in the US (1791 hr/yr/w) but longer than in Europe (eg. UK, 1497 hr/yr/w; France, 1490 hr/yr/w; Germany, 1349 hr/yr/w). Since long working hours are still a leading cause of work-related health problems in Japan, the Japanese Government enacted the Work Style Reform Bill in 2018 to amend 8 laws related to labor conditions [27].

Nevertheless, working hours are directly related to income, and it is necessary to work a certain number of hours to make a living. This truism gives meaning to investigating how work style can be managed in such a way as to maintain sleep quality without markedly reducing working hours. For example, if a worker finishes their job in the early evening, the adverse effect of long working hours on sleep quality might be diminished. In other words, work end time may be an effect modifier for the relationship between working hours and sleep quality. WFH is another candidate effect modifier. To our knowledge, however, no community-based study has examined this question. Regarding a population with specific occupation, there is a review about working hours, sleep and fatigue among workers in public safety sector [16], and the authors stated that only 12% of the identified articles described mitigation strategies or interventions to reduce the adverse effect of long working hours.

Accordingly, we investigated the relationship between working hours and sleep quality among workers recruited from the general population. We then explored work styles which functioned as effect modifiers on the relationship between working hours and sleep quality to suggest appropriate management methods for work style.

## Methods

### Study design and participants

A questionnaire survey was conducted in Ota ward, one of the 23 wards of the Tokyo Metropolitan area, Japan, in cooperation with the local government of the surveyed ward and Toho University. The aim of the survey was to collect information about health, lifestyle, and living/working environment among residents of the ward and to improve local policies. The surveyed ward is located in the southeastern part of Tokyo, and had a population of about 700,000 in October 2021 [28]. The number of business offices and factories in the ward was the sixth-largest in Tokyo in 2016, and 52.2% of employees worked in wholesale/retail businesses, transportation businesses/postal services, or manufacturing industries [29]. In total, 36,000 residents aged in their 20–80s (approximately 5% of the population) were chosen randomly from the residential database in the surveyed ward as participants of the survey.

### Questionnaire and variables

A 14-page questionnaire was distributed to the selected residents by postal mail in September and collected from October to December in 2021, either by postal mail or via an online survey system established by Tokyo Metropolitan government. The questionnaire was assigned an anonymized ID and did not contain personally identifiable questions such as home address or birthday.

The questionnaire enquired about the following information to assess the relationship between working hours and quality of sleep, as well as effect modification by work style. The main exposure was working hours (from more than zero to 20 hours) per day. Information about working hours as well as working start and end time was collected as continuous variables. The outcome was quality of sleep evaluated by the Athens Insomnia Scale (AIS) [30–32], a self-administered psychometric instrument designed for quantifying sleep difficulty based on the 10^th^ revision of the International Classification of Disease criteria. The Japanese version of the AIS has been validated and is widely used in epidemiological studies [14, 33, 34]. The respondents were asked to rate their experience of insomnia-related symptoms over the past month on the following four-point scale: 0 = ‘no problem at all’, 1 = ‘slightly problematic’, 2 = ‘markedly problematic’, 3 = ‘extremely serious problem’. The AIS includes eight items, giving a total score range from 0 to 24. A total score of ≥6 indicates suspected insomnia and consultation with a physician is recommended.

Then, to investigate the influence of some aspects of work style on the relationship between working hours and sleep quality, we chose the following four ‘work styles’ as possible effect modifiers: work end time, shift in working start and end time, current WFH status, and change in work place. Work end time was categorized into 4 groups: ‘12:00–16:59’, ‘17:00–18:59’, ‘19:00–21:59’, and ‘22:00–3:59 (i.e. the next morning)’. Also, we defined four categories of ‘shift in working start and end time’ based on the reply to the following question: ‘How was your working start and end time shifted by the COVID-19 pandemic?’. Answer choices for this question were ‘No change’, ‘Shifted earlier’, ‘Shifted later’, and ‘Became Inconsistent’, and were directly used for categorization. Thirdly, current WFH status was determined by the response to a question regarding the number of WFH days per week (‘How many days do you work from home?’). If the answer was not zero, the respondents were categorized as individuals who worked from home (WFH). Conversely, if the answer was zero or left blank, they were categorized as individuals who did not work from home (No WFH). Lastly, we defined three categories of ‘change in work place’ according to the reply to the following question: ‘How was your work place changed by the COVID-19 pandemic?’ Answer choices were ‘No change’, ‘Work from home (WFM) was newly introduced’, and ‘Proportion of WFM was increased’, and were directly used for categorization.

The questionnaire also collected information about other variables reported as risk factors of insomnia for inclusion in statistical models, namely age (continuous), gender, body mass index (BMI) (<18.5, 18.5–24.9, ≥25.0) [35], comorbidity (no vs. yes), current drinking (no vs. yes), current smoking (no vs. yes), walking time/day (<60 vs. ≥60 minutes/day) [36], educational attainment (‘Vocational school/junior college or lower’ vs. ‘University or graduate school’), and cohabitants (‘living with someone’ vs. ‘living alone’) [15]. BMI was calculated as body weight divided by the square of body height (kg/m^2^). Comorbidity (‘yes’) was defined as having any of the following diseases: hypertension [37], diabetes [38], stroke [39], myocardial infarction/angina [40], cancer [41], liver disease [42], kidney disease [43], gait disturbance, and other diseases. Among ‘other diseases,’ listed in a free description field, allergy and rheumatologic disorders were the most common, following by digestive system and musculoskeletal disorders. Many of these disorders are related to pain to some extent which is known to cause sleep disturbance [44].

### Statistical analysis

**Fig. 1:**
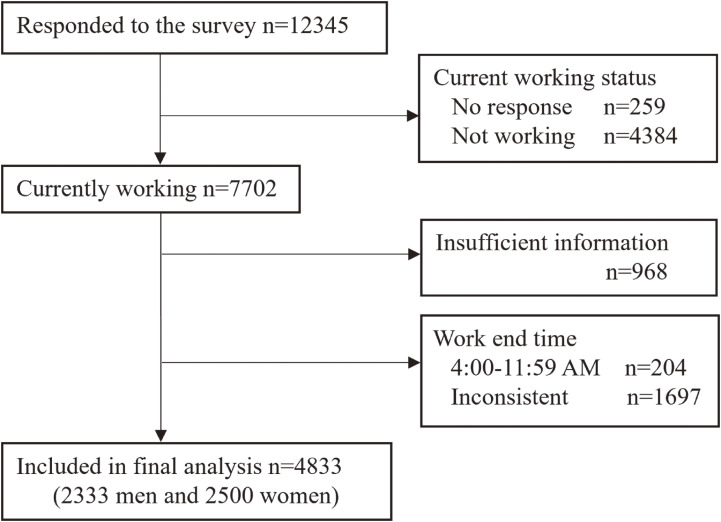
Flow diagram of participant selection for analysis

Among those who received the questionnaire (n = 36,000), 12,345 responses were received (response rate: 34.4%; response by postal mail, 81.8%; via online survey system, 18.2%). Participant selection for the analysis is shown in Fig. 1. Those who answered ‘Yes’ to the question ‘Are you currently working?’ were regarded as current workers (n = 7,702). Respondents who finished work between 4:00 am and 11:59 am and those who answered with an inconsistent work end time were presumed to be shift-time workers with irregular working patterns and not included in the analysis. We also excluded those who did not answer questions which were essential for the analysis. Finally, 4,833 current workers (2,333 men and 2,500 women) were included in the analysis.

Baseline characteristics were described as means, standard deviation (SD)s for continuous variables and n (%) for categorical variables by gender. The relationship between AIS score and major characteristics, including working hours and work styles, was then investigated by gender. Differences in AIS score between categories were tested by the t-test or analysis of variance (ANOVA). If the categories of a variable were in order (i.e. age, working hours, and work end time), trends of association were examined using univariate linear regression models which assigned scores to the level of the independent variable. For working hours, we also conducted univariate linear regression analysis which includes AIS score as a dependent variable and working hours as a continuous independent variable (not shown in tables).

Next, we investigated effect modification by work style on the relationship between working hours and sleep quality. Multivariate linear regression models including AIS score as a dependent variable and working hours as a major independent variable were used. Any of ‘work end time’, ‘shift in working start and end time’, ‘current WFH status’, or ‘change in work place’ was included in the multivariate models with interaction terms such as [‘working hours’ × ‘work end time’]. Since the categories of ‘work end time’ were in order, trends of interaction were examined using regression models which assigned scores to the order of the ‘work end time’ variable in an interaction term, including the same sets of covariates. The effect modifications are depicted in Supplementary figures along with tables. The covariates included in the models are described in the subsection on the questionnaire and variables, i.e., age, gender, BMI, comorbidity, current drinking, current smoking, walking hours, educational attainment, and cohabitants.

All analyses were performed with Stata/MP 16 for Windows (Stata Corp LLC, Texas, USA). Statistical tests were two-sided, and p values of <0.05 were considered statistically significant.

## Results

### Participant characteristics and sleep quality

Characteristics of the participants are shown in Table 1. Average age of the total population was 48.6 years. Average AIS score was 5.0, and average working hours was 8.0 hours/day. AIS scores by category of working condition and basic characteristics are shown in Table 2. A higher AIS score, i.e., lower sleep quality, was observed in younger participants, those with comorbidities, those with shorter walking hours, and those without cohabitants. Also, longer working hours, later work end time, later working start and end time, and WFH groups were significantly associated with lower sleep quality. Regarding longer working hours, a univariate linear regression model which included working hours as a continuous variable showed the same association (β = 0.17, 95% confidence interval (CI) [0.12, 0.22], not shown in Table 2). As trends in men and women were similar, except for WFH, we conducted further analysis with both genders combined.

**Table 1:** Characteristics of participants

**Variable**	**Category**	**n (%) or mean, SD**					

		**Total**		**Men**		**Women**	
		**n = 4833**		**n = 2333**		**n = 2500**	
Age (continuous)		48.6	, 14.1	50.6	, 14.2	46.7	, 13.7

Age (years)	20–39	1383	(28.6)	572	(24.5)	811	(32.4)
	40–49	1115	(23.1)	514	(22.0)	601	(24.0)
	50–59	1235	(25.6)	580	(24.9)	655	(26.2)
	60–89	1100	(22.8)	667	(28.6)	433	(17.3)

AIS score		5.0	, 3.6	4.9	, 3.6	5.2	, 3.6
Sleep duration (hrs/day)		7.0	, 1.2	7.0	, 1.2	6.9	, 1.2
Working hours (hrs/day)		8.0	, 2.0	8.6	, 1.8	7.4	, 1.9

Working hours (hrs/day)	<5	301	(6.2)	57	(2.4)	244	(9.8)
	5–<8	1274	(26.4)	364	(15.6)	910	(36.4)
	8–<10	2907	(60.2)	1645	(70.5)	1262	(50.5)
	≥11	351	(7.3)	267	(11.4)	84	(3.4)

Work end time (24-hour clock time)	12:00–16:59	858	(17.8)	250	(10.7)	608	(24.3)
	17:00–18:59	2429	(50.3)	1156	(49.6)	1273	(50.9)
	19:00–21:59	1381	(28.6)	820	(35.2)	561	(22.4)
	22:00–3:59	165	(3.4)	107	(4.6)	58	(2.3)

Shift in working start and end time	No change	4005	(82.9)	1907	(81.7)	2098	(83.9)
	Shifted earlier	435	(9.0)	240	(10.3)	195	(7.8)
	Shifted later	180	(3.7)	92	(3.9)	88	(3.5)
	Became inconsistent	213	(4.4)	94	(4.0)	119	(4.8)

Current WFH status	No WFH	2924	(60.5)	1305	(55.9)	1619	(64.8)
	WFH	1909	(39.5)	1028	(44.1)	881	(35.2)

Change in work place^a^	No change	2981	(61.7)	1333	(57.1)	1648	(65.9)
	WFH newly introduced	1227	(25.4)	636	(27.3)	591	(23.6)
	Proportion of WFH increased	625	(12.9)	364	(15.6)	261	(10.4)

Body mass index	<18.5	448	(9.3)	66	(2.8)	382	(15.3)
	18.5–24.9	3373	(69.8)	1570	(67.3)	1803	(72.1)
	≥25.0	1012	(20.9)	697	(29.9)	315	(12.6)

Comorbidity^b^	No	3226	(66.8)	1399	(60.0)	1827	(73.1)
	Yes	1607	(33.3)	934	(40.0)	673	(26.9)

Current drinking	No	1665	(34.5)	597	(25.6)	1068	(42.7)
	Yes	3168	(65.6)	1736	(74.4)	1432	(57.3)

Current smoking	No	4176	(86.4)	1869	(80.1)	2307	(92.3)
	Yes	657	(13.6)	464	(19.9)	193	(7.7)

Walking hours (min/day)	<60	3075	(63.6)	1594	(68.3)	1481	(59.2)
	≥60	1758	(36.4)	739	(31.7)	1019	(40.8)

Educational attainment	Vocational school/ Junior college or lower	2172	(44.9)	807	(34.6)	1365	(54.6)
	University or graduate school	2661	(55.1)	1526	(65.4)	1135	(45.4)

Cohabitants	No (living alone)	927	(19.2)	415	(17.8)	512	(20.5)
	Yes	3906	(80.8)	1918	(82.2)	1988	(79.5)

Abbreviation: AIS, Athens Insomnia Scale; SD, standard deviation; WFH, work from home

^a^Change in work place is defined by answers for the following question: ‘How was your work place changed by the COVID-19 pandemic?’.

^b^Comorbidity was defined as having any one of the following diseases: hypertension, diabetes, stroke, myocardial infarction/angina, cancer, liver disease, kidney disease, gait disturbance, and others.

**Table 2:** Relationship between AIS and working conditions/major characteristics by gender

**Variable**	**Category**	**AIS score**					

		**Total**		**Men**		**Women**	
		**n = 4833**		**n = 2333**		**n = 2500**	
	
		**mean, SD**	**p value^a^**	**mean, SD**	**p value^a^**	**mean, SD**	**p value^a^**
Age (years)	20–39	5.3, 3.6	<0.01	5.1, 3.5	<0.01	5.4, 3.7	0.03
	40–49	5.0, 3.5		5.0, 3.7		5.0, 3.4	
	50–59	5.2, 3.6		5.1, 3.7		5.3, 3.5	
	60–89	4.5, 3.5		4.4, 3.4		4.8, 3.5	

Working hours (hrs/day)	<5	4.5, 3.5	<0.01	4.0, 4.0	<0.01	4.6, 3.3	<0.01
	5–<8	4.8, 3.5		4.5, 3.5		4.9, 3.5	
	8–<10	5.0, 3.5		4.8, 3.5		5.4, 3.5	
	≥11	6.1, 3.9		5.9, 3.7		7.0, 4.3	

Work end time (24-hour clock time)	12:00–16:59	4.7, 3.4	<0.01	4.4, 3.5	<0.01	4.8, 3.4	<0.01
	17:00–18:59	5.0, 3.6		4.7, 3.6		5.2, 3.6	
	19:00–21:59	5.2, 3.5		5.1, 3.4		5.4, 3.5	
	22:00–3:59	6.3, 4.1		6.2, 3.9		6.3, 4.5	

Shift in working start and end time	No change	4.9, 3.5	<0.01	4.7, 3.5	<0.01	5.0, 3.5	<0.01
	Shifted earlier	5.4, 3.8		5.1, 3.8		5.8, 3.7	
	Shifted later	6.3, 3.6		6.4, 3.5		6.1, 3.6	
	Became inconsistent	5.8, 4.0		5.5, 3.7		6.1, 4.2	

Current WFH status	No WFH	5.0, 3.5	0.26	4.8, 3.6	0.98	5.1, 3.5	0.05
	WFH	5.1, 3.6		4.9, 3.6		5.4, 3.7	

Change in work place^b^	No change	4.9, 3.5	0.03	4.8, 3.6	0.44	5.0, 3.5	0.02
	WFH newly introduced	5.2, 3.6		5.0, 3.5		5.4, 3.7	
	Proportion of WFH increased	5.2, 3.7		4.9, 3.7		5.5, 3.7	

Body mass index	<18.5	5.3, 3.6	0.01	5.2, 3.4	0.23	5.3, 3.7	<0.01
	18.5–24.9	4.9, 3.5		4.8, 3.5		5.1, 3.5	
	≥25.0	5.2, 3.8		5.0, 3.8		5.7, 3.9	

Comorbidity^c^	No	4.8, 3.4	<0.01	4.7, 3.5	<0.01	5.0, 3.4	<0.01
	Yes	5.4, 3.8		5.1, 3.7		5.7, 3.8	

Current drinking	No	5.1, 3.6	0.08	4.9, 3.6	0.70	5.3, 3.5	0.30
	Yes	5.0, 3.6		4.8, 3.5		5.1, 3.6	

Current smoking	No	5.0, 3.5	0.47	4.9, 3.6	0.72	4.8, 3.6	0.07
	Yes	5.1, 3.8		4.9, 3.6		4.9, 3.6	

Walking hours (min/day)	<60	5.2, 3.6	<0.01	5.1, 3.6	<0.01	5.4, 3.7	<0.01
	≥60	4.7, 3.4		4.3, 3.3		4.9, 3.4	

Educational attainment	Vocational school/Junior college or lower	5.1, 3.7	0.09	4.9, 3.9	0.47	5.2, 3.6	0.44
	University or graduate school	4.9, 3.5		4.8, 3.4		5.1, 3.6	

Cohabitants	No (living alone)	5.6, 3.7	<0.01	5.6, 3.7	<0.01	5.6, 3.7	<0.01
	Yes	4.9, 3.5		4.7, 3.5		5.1, 3.5	

Abbreviation: AIS, Athens Insomnia Scale; SD, standard deviation, WFH; work from home

^a^Differences of the AIS score between categories were tested by the t test for binary variables or ANOVA for categorical variables. If the categories of a variable were in order (i.e. age, working hours, and work end time), trends of association were examined using univariate linear regression models which assigned scores to the level of the independent variable.

^b^Change in work place is defined by the answer to the following question: ‘How was your work place changed by the COVID-19 pandemic?’

^c^Comorbidity was defined as having any one of the following diseases: hypertension, diabetes, stroke, myocardial infarction/angina, cancer, liver disease, kidney disease, gait disturbance, and others.

### Effect modifications by work style

We then investigated effect modification by work style on the relationship between working hours and sleep quality by multivariate linear regression analysis which included an interaction term for each work style. Results for work end time are shown in Table 3 and Supplementary Fig. 1. After adjusting for possible confounding factors, the magnitude of the relationship between long working hours and sleep quality was significantly larger when work end time was later (p for trend of interaction < 0.01, Supplementary Fig. 1). Similarly, when the working start and end time shifted later, the magnitude of the relationship between long working hours and sleep quality was significantly greater than in the ‘no change’ group (interaction term, β = 0.32, 95% CI [0.09, 0.55]) as shown in Table 4 and Supplementary Fig. 2. Further, effect modification by current WFH status and change in work place was examined using the same regression models (Table 5 and Supplementary Fig. 3, Table 6 and Supplementary Fig. 4, respectively). There was no significant effect modification by current WFH status (interaction term, β = 0.06, 95% CI [−0.06, 0.17]). However, when the proportion of WFH was increased, the magnitude of the relationship between long working hours and sleep quality tended to be larger than in the ‘no change’ group, albeit that the interaction was not statistically significant (interaction term, β = 0.16, 95% CI [−0.01, 0.34]).

**Table 3:** Relationship between working hours and AIS: effect modification by work end time

**Variable**	**Category**	**n = 4833**	
		**β^a^**	**95% CI**
Working hours (hrs/day)		0.14	[0.02, 0.26]*

Work end time (24-hour clock time)	12:00–16:59	0.34	[−0.92, 1.60]
	17:00–18:59	ref	
	19:00–21:59	−0.59	[−1.99, 0.80]
	22:00–3:59	−0.72	[−2.73, 1.28]

Working hours × Work end time (interaction term)^b^	12:00–16:59	−0.06	[−0.25, 0.12]
	17:00–18:59	ref	
	19:00–21:59	0.08	[−0.09, 0.24]
	22:00–3:59	0.18	[−0.03, 0.38]

Abbreviation: AIS, Athens Insomnia Scale

*p < 0.05

^a^To assess effect modification by work end time on the relationship between working hours and AIS score, a multivariate linear regression model including AIS score as a dependent variable and working hours as a major independent variable was used. In this model, work end time was included with the interaction term as [working hours × work end time]. Included covariates are age, gender, body mass index, comorbidity, current drinking, current smoking, walking hours, educational attainment, and cohabitants.

^b^Since the categories of ‘work end time’ was in order, the trend of interaction was examined using a regression model which assigned scores to the order of ‘work end time’ variable in an interaction term, including the same sets of covariates. The trend of interaction was significant (p < 0.01).

**Table 4:** Relationship between working hours and AIS: effect modification by shift in working start and end time

**Variable**	**Category**	**n = 4833**	
		**β^a^**	**95% CI**
Working hours (hrs/day)		0.17	[0.11, 0.24]*

Shift in working start and end time	No change	ref	
	Shifted earlier	1.15	[−0.58, 2.87]
	Shifted later	−1.71	[−3.81, 0.39]
	Became inconsistent	0.25	[−1.71, 2.20]

Working hours × Shift in working start and end time (interaction term)	No change	ref	
	Shifted earlier	−0.09	[−0.29, 0.12]
	Shifted later	0.32	[0.09, 0.55]*
	Became inconsistent	0.09	[−0.16, 0.33]

Abbreviation: AIS, Athens Insomnia Scale

*p < 0.05

^a^To assess effect modification by shift in working start and end time on relationship between working hours and AIS score, a multivariate linear regression model including AIS score as a dependent variable and working hours as a major independent variable was used. In this model, shift in working start and end time was included with the interaction term as [working hours × shift in working start and end time]. Included covariates are age, gender, body mass index, comorbidity, current drinking, current smoking, walking hours, educational attainment, and cohabitants.

**Table 5:** Relationship between working hours and AIS: effect modification by current WFH status

**Variable**	**Category**	**n = 4833**	
		**β^a^**	**95% CI**
Working hours (hrs/day)		0.18	[0.12, 0.25]*

Current WFH status^b^	No WFH	ref	
	WFH	−0.49	[−1.46, 0.48]

Working hours × Current WFH status (interaction term)	No WFH	ref	
	WFH	0.06	[−0.06, 0.17]

Abbreviation: AIS, Athens Insomnia Scale; WFH, work from home

*p < 0.05

^a^To assess the effect modification by current WFH status on the relationship between working hours and AIS score, a multivariate linear regression model including AIS score as a dependent variable and working hours as a major independent variable was used. In this model, current WFH status was included with the interaction term as [working hours × current WFH status]. Included covariates are age, gender, body mass index, comorbidity, current drinking, current smoking, walking hours, educational attainment, and cohabitants.

^b^Current WFH status was defined by the answer to the following question: ‘How many days do you work from home?’. If the answer was not zero, the respondents were categorized as individuals who worked from home (WFH). Conversely, if the answer was zero or left blank, they were categorized as individuals who did not work from home (No WFH).

**Table 6:** Relationship between working hours and AIS: effect modification by change of work place

**Variable**	**Category**	**n = 4833**	
		**β^a^**	**95% CI**
Working hours (hrs/day)		0.18	[0.11, 0.24]*

Change in work place	No change	ref	
	WFH newly introduced	0.11	[−1.08, 1.31]
	Proportion of WFH increased	−1.26	[−2.76, 0.25]

Working hours × Change in work place^b^ (interaction term)	No change	ref	
	WFH newly introduced	−0.001	[−0.14, 0.14]
	Proportion of WFH increased	0.16	[−0.01, 0.34]

Abbreviation: AIS, Athens Insomnia Scale; WFH, work from home

*p < 0.05

^a^To assess the effect modification by change in work place on the relationship between working hours and AIS score, a multivariate linear regression model including AIS score as a dependent variable and working hours as a major independent variable was used. In this model, change in work place was included with the interaction term as [working hours × change in work place]. Included covariates are age, gender, body mass index, comorbidity, current drinking, current smoking, walking hours, educational attainment, and cohabitants.

^b^Change in work place was defined by the answer to the following question: ‘How was your work place changed by the COVID-19 pandemic?’.

## Discussion

In this study, we firstly assessed the relationship between working hours and sleep quality. Longer working hours were significantly associated with lower sleep quality among workers recruited from a general population. Then, we investigated effect modification by work style on the relationship between working hours and sleep quality. The later the work end time was, the greater the magnitude of the relationship between longer working hours and lower sleep quality was. Similarly, when the working start and end time shifted later, the magnitude of the relationship between longer working hours and lower sleep quality was greater compared to the no change group. Although no effect modification by current WFH status was observed, the magnitude of the relationship between longer working hours and lower sleep quality also tended to become larger when the proportion of WFH was increased, compared with the no change group.

In line with previous studies conducted in workers of the same occupation [13, 14, 45], our results showed that longer working hours were significantly associated with lower sleep quality even among workers in various occupations. For example, Hori et al. found that Japanese public school teachers with longer working hours (from 50 hours to ≥80 hours per week) had lower sleep quality as evaluated by AIS [14]. In a cohort of civil servants in London, Virtanen et al. indicated that long working hours predict the onset of new sleep disturbances [10]. Based on our results for workers in various occupations, we can state that the relationship between longer working hours and lower sleep quality is universally observed irrespective of age, gender, or job type.

Our study indicated that the magnitude of the relationship between longer work hours and lower sleep quality increased as work end time became later. The same effect modification was observed when the working start and end time shifted later. Although several studies have examined the association between long working hours and sleep quality in day-time workers [13, 17], none have considered working style as effect modifiers, such as work end time or WFH. One previous study focused on work end time of airline short-haul pilots [46], but examined the direct effects of later working time. Results showed that fatigue level was more severe among those on late-finishing duties compared with those working in an earlier shift even when flight duration was the same. Most other studies about the effect of working late have focused on shift work [22], or night shift in limited occupations such as healthcare workers [47, 48]. Our present study newly indicates that leaving work earlier may diminish the magnitude of the relationship between longer working hours and lower sleep quality even in regular day-time workers. In other words, the beneficial effect of finishing work earlier on sleep quality was larger in those whose working hours were longer. We also speculate that allowing an earlier return home on a few days per week may be effective in maintaining sleep quality, because in addition to our finding that earlier work end time was effective, we also found that the magnitude of the relationship between working hours and sleep quality was the same between those whose working start and end time became inconsistent and those whose working start and end time did not change.

Despite the current WFH status did not act as an effect modifier, we found that when the proportion of WFH increased, the magnitude of the relationship between longer working hours and lower sleep quality tended to become larger compared with the no change group. In contrast, newly introduced WFH did not function as an effect modifier. Although the effects of WFH on health and its mechanism are still inconclusive, advantages of WFH reported to date include reduced commuting time, high job control [49], control of work environmental factors [50], and avoiding distraction from other colleagues [51]. Conversely, Xiao et al [52] stated that decreased physical activity, social isolation, and lack of communication with coworkers were significant predictors of decreased physical and mental health status. As mentioned above, only a few studies have evaluated the direct effect of WFH on sleep quality. While Çiftçi reported that sleep quality did not differ between office workers and those who work from home [24], Lee et al found that WFH was significantly associated with multiple insomnia symptoms compared with a conventional work group [25]. In our study, since workers who first experienced WFH may have been affected by both beneficial and harmful aspects of WFH, newly introduced WFH did not act as effect modifier for the relationship between working hours and sleep quality. However, an increased proportion of WFH might have caused more harmful effects such as social isolation or decreased physical activity, which might in turn have had detrimental effects of long working hours on sleep quality. Miyake et al. reported similar results, namely that workers who worked remotely 4 or more days per week (i.e., those who frequently work remotely) were marginally more likely to report feeling lonely compared with those who did not work remotely [53]. Optimization of the work environment for those who often work from home is an important topic, and may decrease sleep problems due to long working hours.

Our study had several strengths. First, the survey participants were selected randomly from the basic resident register of a ward in Tokyo. Accordingly, we assume that the relationship between working hours and sleep quality is applicable not only within certain occupations but also across various occupations, including small and medium-sized businesses. Optimization of working style must be universally effective in diminishing the adverse effects of long working hours on sleep quality. Second, sleep quality was quantitatively assessed using AIS score, which has been validated [30]. Third, the study contained both information about time in length (i.e. working hours) and clock time (i.e. work end time as well as shift in working start and end time). This enabled us to understand the effect of not only the length of work but also the time at which to end work on decreasing the adverse effect of long working hours.

However, several limitations of this study should be mentioned. First, the study was conducted in one ward in Tokyo and participation rate was 34.4%. Participants in this kind of study are thought to be more health-conscious, and/or more likely to have enough time to answer the questionnaire than the general population. A similar tendency has been reported in previous studies [54, 55]. Regarding our study, a higher response rate would have included more participants with worse conditions, such as poorer sleep quality, longer working hours, or working later. This might have led to a greater magnitude in the relationship between working hours and sleep quality than we actually observed. Second, this study was focused on day-time workers, and excluded those thought to be shift workers. Our results are therefore not applicable to shift workers. Also, since all participants lived in an urban area in Tokyo, the results might not be applicable to workers in rural areas. However, the analyzed participants were not limited by occupation, which would ensure at least some degree of generalizability of the results. Third, the questionnaire did not directly collect information on whether participants were shift workers; instead, we defined shift workers by their work end time. This may have resulted in a degree of misclassification and inappropriate exclusion of participants from the analysis. In particular, participants who answered that their work end time was inconsistent were deemed as shift workers and excluded, even if they were not shift workers. We cannot specify this type of misclassification, but if it were non-differential and more participants were included in the analysis, we might have been able to observe the association more definitively. Also, our questionnaire didn’t contain any items to directly categorize the participants to regular (full-time)/irregular (part-time) workers. However, it is possible that work end time is related to this working condition. Thus, instead of the categories showing regular/irregular workers, we used number of working days per week as a surrogate variable and performed an additional analysis to examine whether there was effect modification by the number of working days (≥5 days or <5 days per week). There was no significant effect modification by working days per week on the relationship between working hours and sleep quality (interaction term, β = −0.09, 95% CI [−0.29, 0.12]. not shown in the results). Fourth, since there were less long-time workers in the group of earliest work end time group, effect modification by work end time should be carefully interpreted, particularly in the range of extremely long working hours. Long-time workers tended to finish their work late. Fifth, although many confounding factors were adjusted in the analysis, residual confounding factors may have been present, such as cohabitation with infants, occupation, and economic conditions. Instead of economic conditions, we adopted educational attainment, which is strongly related to economic status, as a covariate [56]. Lastly, due to the cross-sectional design of the study, the direction of causality could not be specified. However, irrespective of the direction of causality between longer working hours and lower sleep quality, this relationship is undesirable. Therefore, we consider that leaving work earlier and/or optimizing the environment of WFH are meaningful in maintaining good sleep quality, in addition to appropriate working hours.

## Conclusions

Although the Work Style Reform Bill has been enacted in 2018 to ameliorate working conditions including long working hours, longer working hours were significantly associated with lower sleep quality in day-time workers living in Tokyo. Later work end time, later working start and end time, and a higher proportion of WFH strengthened this relationship as effect modifiers. Leaving work earlier and optimizing the work environment at home may be effective in securing good sleep quality.

## Abbreviations

AIS: Athens Insomnia Scale
BMI: body mass index
CI: confidence interval
WFH: work from home

## Supplementary information

**Additional file 1:** **Supplementary figure 1** Relationship between working hours and AIS with consideration to interaction by work end time. **Supplementary figure 2** Relationship between working hours and AIS with consideration to interaction by shift in working start and end time. **Supplementary figure 3** Relationship between working hours and AIS with consideration to interaction by current WFH status. **Supplementary figure 4** Relationship between working hours and AIS with consideration to interaction by change in work place.

## Declarations

### Availability of data and materials

The data analyzed in this study are not publicly available.
