# Supplementary material for: Relationship between working hours and sleep quality with consideration to effect modification by work style: a community-based cross-sectional study
Source: Environ Health Prev Med. 2024 Mar 20;29:19. doi: 10.1265/ehpm.23-00252 (PMC10965413; doi:10.1265/ehpm.23-00252)
Supplement: Supplementary file 1 — Additional file 1: Supplementary figure 1 Relationship between working hours and AIS with consideration to interaction by work end time. Supplementary figure 2 Relationship between working hours and AIS with consideration to interaction by shift in working start and end time. Supplementary figure 3 Relationship between working hours and AIS with consideration to interaction by current WFH status. Supplementary figure 4 Relationship between working hours and AIS with consideration to interaction by change in work place. [file ehpm-29-019-s001.docx]

**Supplementary information**

**Additional file 1:**


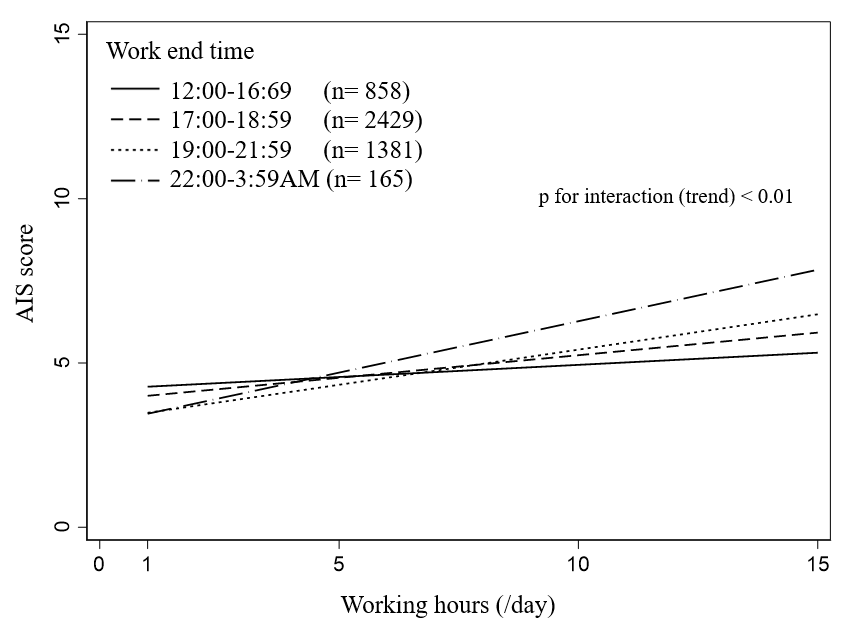


**Supplementary figure 1** Relationship between working hours and AIS with consideration to interaction by work end time

Predicted relationships between working hours and AIS score by category of work end time are shown. Age, gender, body mass index, comorbidity, current drinking, current smoking, walking hours, educational attainment, and cohabitants were adjusted in the linear regression model.

AIS: Athens Insomnia Scale.

**
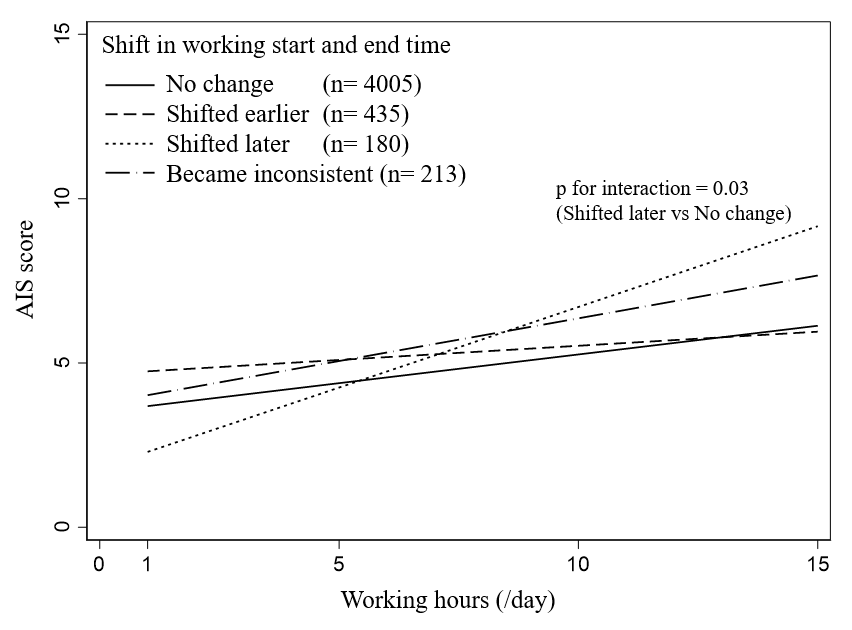
Supplementary figure 2** Relationship between working hours and AIS with consideration to interaction by shift in working start and end time

Predicted relationships between working hours and AIS score by category of shift in working start and end time are shown. Age, gender, body mass index, comorbidity, current drinking, current smoking, walking hours, educational attainment, and cohabitants were adjusted in the linear regression model.

AIS: Athens Insomnia Scale.

**
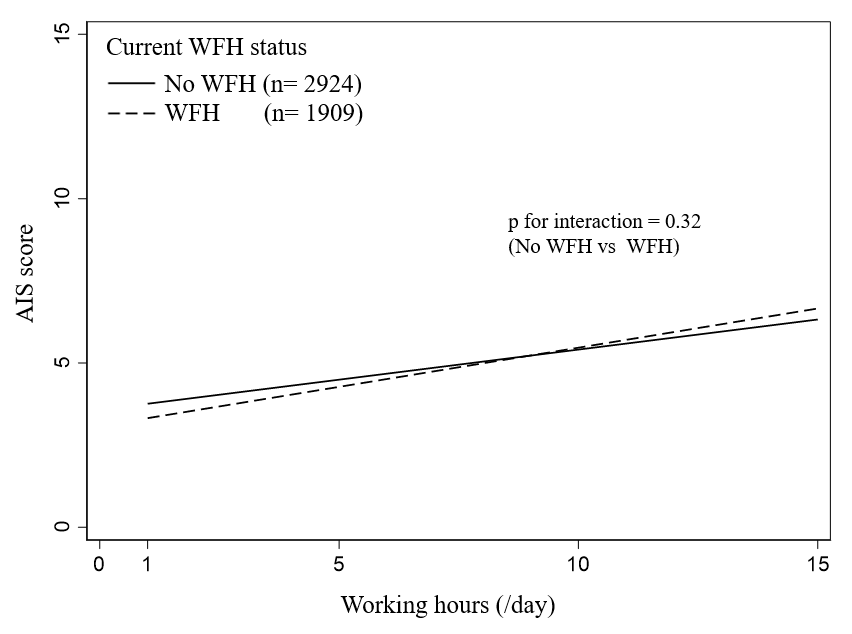
Supplementary figure 3** Relationship between working hours and AIS with consideration to interaction by current WFH status

Predicted relationships between working hours and AIS score by category of current WFH status are shown. Age, gender, body mass index, comorbidity, current drinking, current smoking, walking hours, educational attainment, and cohabitants were adjusted in the linear regression model.

AIS: Athens Insomnia Scale; WFH: work from home

**
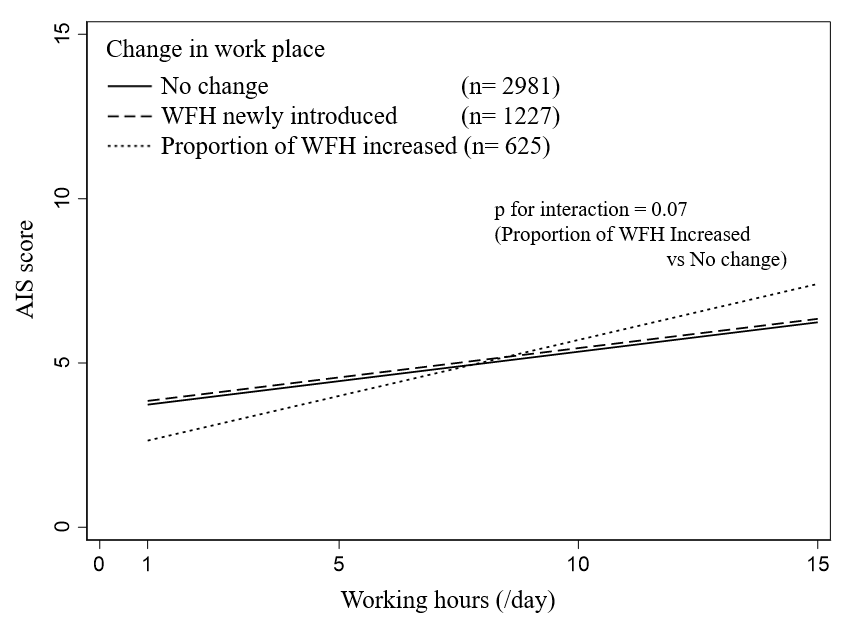
Supplementary figure 4** Relationship between working hours and AIS with consideration to interaction by change in work place

Predicted relationships between working hours and AIS score by category of change in work place are shown. Age, gender, body mass index, comorbidity, current drinking, current smoking, walking hours, educational attainment, and cohabitants were adjusted in the linear regression model.

AIS: Athens Insomnia Scale; WFH: work from home
